# Predicting Sustainable Employability in Swedish Healthcare: The Complexity of Social Job Resources

**DOI:** 10.3390/ijerph17041200

**Published:** 2020-02-13

**Authors:** Marta Roczniewska, Anne Richter, Henna Hasson, Ulrica von Thiele Schwarz

**Affiliations:** 1 Procome Research Group, Medical Management Centre, Department of Learning, Informatics, Management and Ethics, Karolinska Institutet, 171 76 Stockholm, Sweden; anne.richter@ki.se (A.R.); henna.hasson@ki.se (H.H.); ulrica.schwarz@ki.se (U.v.T.S.); 2 Sopot Campus, SWPS University of Social Sciences and Humanities, 81-745 Sopot, Poland; 3 Centre for Epidemiology and Community Medicine, 104 31 Stockholm, Sweden; 4 School of Health, Care and Social Welfare, Mälardalen University, 722 20 Västerås, Sweden

**Keywords:** sustainable employability, healthcare, female-dominated workplace, social job resources, trust, teamwork, job satisfaction, health, job performance

## Abstract

Achieving sustainable employability (SE), i.e., when employees are able to continue working in a productive, satisfactory, and healthy manner, is a timely challenge for healthcare. Because healthcare is a female-dominated sector, our paper investigated the role of social job resources in promoting SE. To better illustrate the complexity of the organizational environment, we incorporated resources that operate at different levels (individual, group) and in different planes (horizontal, vertical): trust (individual-vertical), teamwork (group-horizontal), and transformational leadership (group-vertical). Based on the job demands-resources model, we predicted that these resources initiate the motivational process and thus promote SE. To test these predictions, we conducted a 3-wave study in 42 units of a healthcare organization in Sweden. The final study sample consisted of 269 professionals. The results of the multilevel analyses demonstrated that, at the individual level, vertical trust was positively related to all three facets of SE. Next, at the group level, teamwork had a positive link with employee health and productivity, while transformational leadership was negatively related to productivity. These findings underline the importance of acknowledging the levels and planes at which social job resources operate to more accurately capture the complexity of organizational phenomena and to design interventions that target the right level of the environment.

## 1. Theoretical Introduction

### 1.1. The Importance of Sustainable Employability in Healthcare

With the aging of the workforce and many individuals leaving the labor market for health reasons, sustainable employability (SE) is a growing concern for many societies [1,2]. SE is defined as the opportunity for workers to “make a valuable contribution through their work, now and in the future, while safeguarding their health and welfare” [3] (p. 74). From an organizational perspective, a sustainable workforce is vital because it reduces the costs of turnover and absenteeism caused by employee ill health and reduced productivity. SE is especially important in healthcare services, as it supports the retention of trained specialists who are difficult to replace because of their education, skills, and expertise. Yet, the problem is that turnover is relatively high in healthcare settings. For example, a prospective questionnaire study of healthcare managers in Sweden demonstrated that 40% left their jobs within a 4-year period [4]. Such high turnover bears negative consequences; for example, it causes instability in the employee group, creates an increased workload for the remaining staff, and is also linked with poorer quality patient care [5,6]. Thus, the need for a stable and healthy workforce within the healthcare sector calls for the urgent investigation of factors that promote SE in this environment for the benefit of employees, organizations, patients, and the economy of many countries. SE means that workers are able to continue working productively throughout their lives while retaining their health and well-being [3]. Thus, SE does not only entail retaining workers’ productivity. The definition implies that SE has three equally important facets: apart from *productivity*, SE includes good *mental and physical health* and *well-being at work* (e.g., job satisfaction). In this paper, we follow a growing paradigm shift in occupational health, where the focus is diverted from work as a risk factor for ill health to work as a source of vitality, empowerment, and healthy aging [1]. While many studies have focused on causes for burnout [7] or high turnover intentions among nurses and doctors [8], it is questionable whether these causes of ill health and turnover are the same as the factors that promote a stable and healthy workforce. In fact, Linberg and colleagues [9] demonstrated that more than half of the determinants they investigated were associated solely with *either* promoting excellent work ability (e.g., feedback) *or* with preventing poor work ability (e.g., job security). This pattern of results allows us to argue that separate factors may be responsible for SE than those that affect turnover or absenteeism. Thus, we contribute to the literature by shifting the interest towards factors that *promote* SE rather than focusing on those that inhibit it. We do so by applying a multilevel and multidirectional perspective on how social job resources shape SE in healthcare.

### 1.2. Social Job Resources

In most countries, healthcare is a female-dominated sector [10]. The literature generally supports the idea that women are more relational than men [11] and are more likely than men to value dyadic bonds rather than categorical group affiliations [12]. For example, in a study among U.S. postal employees, women used prosocial coping in response to stress more often than men [13]. The preponderance of women in a workplace is likely to shape the characteristics of this environment, i.e., job demands and job resources that are available. Since women tend to place more emphasis on relationships than men, it is probable that social job resources are particularly important in this environment. Indeed, a recent integrative review identified six key job resources of nursing staff and four of them related to social aspects of the workplace [14]. Moreover, a scoping review pointed to the significance of social processes for the retention of health workforces [15].

According to the job demands-resources (JD-R) model, *job resources* refer to those physical, psychological, social, or organizational aspects of the job that help employees reach any of the following objectives: (a) manage their job demands and the associated strain more effectively, (b) achieve goals at work, and (c) stimulate personal growth [16,17]. *Social job resources* constitute a subcategory of job resources [18]. These resources arise as a possibility because the workplace is a social environment in which individuals function among others, have different levels of interdependence and interact with each other. Being a part of the social matrix allows individuals to benefit from access to group resources. Social job resources comprise resources that can be provided by or obtained from other individuals in the workplace. Examples include emotional and instrumental support, teamwork and collaboration, and opportunities for feedback or mentoring.

Social job resources serve the same function as other types of job resources, i.e., they buffer against the negative effects of job demands, help attain goals, and stimulate professional development [16,19]. The last two points make it evident that resources are not only functional in dealing with job demands, but they also have an inherent motivational quality. This aspect of job resources results from the fact that they fulfill basic human needs for autonomy, competence, and relatedness [20]. Indeed, a recent dominance analysis of the data from the sixth European Working Conditions Survey showed that job resources explained 94%–95% of the variance observed in work engagement [21]. The abundance of job resources triggers a *motivational process*: through improved work engagement, job resources lead to positive outcomes, such as stronger organizational commitment and better work performance [16,19]. For example, individuals who receive more positive reinforcement and who feel empowered perform better [22]. Schaufeli and Bakker [22] found that performance feedback, social support, and supervisory coaching are positively linked with work engagement. Moreover, social job resources support learning and development: collaboration has been found to be an important factor for teachers’ professional learning [23]. These studies support the existence of a direct link between social job resources and employee motivation. The fact that social job resources have intrinsic motivational potential allows them to be studied as predecessors of work ability.

### 1.3. Multilevel and Multidirectional Influences in the Workplace

In the workplace, social job resources operate at multiple levels. These resources may be located at the level of the organization or department (e.g., procedural fairness), in interpersonal relations (e.g., team climate), in the organization of work (e.g., participation in decision making), and at the level of the task (e.g., performance feedback). Individual employees differ with respect to the social job resources that they have access to, and distinct teams or departments share different pools of social resources. Acknowledging these within-group similarities and possible between-group differences allows for the treatment of resources as belonging to a group level. This conceptualization creates the possibility of comparing different groups’ working environments and exploring how these differences influence individual employees. Thus, going beyond individuals’ perceptions and acknowledging many layers of organizational structures allows for a deeper understanding of the complexities in organizational life. Moreover, social job resources may be exchanged in horizontal (colleague–colleague) and vertical (supervisor–subordinate) relations, and it is vital to recognize which of those axes play a more pivotal role in employee well-being and productivity. 

Given the before-mentioned complexity in which employees can exchange resources, we have chosen to investigate three social job resources (vertical trust, teamwork, and transformational leadership), which operate at distinct levels of organizational structure and encompass both vertical and horizontal relations. *Vertical trust* refers to the employee belief in the integrity and credibility of the management and the perceived fairness of organizational conduct [24,25]. It captures the relation between an individual and the organization in a vertical direction, encompassing an individual’s confidence in the fairness and credibility of the organizational conduct. Thus, vertical trust is located at the individual level. *Teamwork* represents how well people cooperate with each other and structure their work in a workgroup [26]. It is a horizontal exchange of resources (e.g., knowledge, practical support) between employees in a certain workgroup, and this pool of resources is shared by its members. Therefore, this resource is located at the group level and is exchanged horizontally. Finally, *transformational leadership* refers to the leader motivating his or her followers beyond immediate self-interests through idealized influence, inspirational motivation, intellectual stimulation, or individualized consideration [27]. Because leadership is a process of influencing the activities of a group in efforts towards goal achievement [28], it can be viewed as a social job resource that is exchanged in a vertical direction between the manager and the group. Therefore, we treat transformational leadership as a group-level social resource, acting in a vertical plane between the leader and the followers.

### 1.4. The Present Study

In this investigation, we apply the JD-R model [19], which argues that job resources launch the motivational process and thus have a positive influence on employees. We predict that social job resources, i.e., vertical trust, teamwork, and transformational leadership, are positively linked with the three facets of SE: job satisfaction, health, and job performance. Previous studies from the healthcare sector provide grounds for these hypotheses. Social capital (including vertical trust) among healthcare professionals predicted their job satisfaction and work engagement [29], and job satisfaction mediated the relationship between vertical trust and distributed leadership, which had a further positive impact on job performance [30]. Furthermore, a higher level of teamwork among nurses was linked with greater job satisfaction [31], and the findings of a systematic review showed how good teamwork was linked with better patient safety [32]. Finally, the importance of transformational leadership for healthcare employees’ productivity [33] and well-being [34] has been recognized. While parts of the hypothesized relationships have been tested, the knowledge gap concerns how these social job resources work jointly in vertical and horizontal planes when they are framed in a multilevel perspective. To the best of our knowledge, no previous research has attempted to investigate the relative roles of these three types of social job resources in shaping SE. Yet, in organizational settings, these resources act simultaneously; therefore, simultaneously including these multiple influences mirrors the organizational context more truly. It is also likely that different social job resources may be more relevant for distinct facets of SE. We treat this latter matter as exploratory in this investigation.

We set out to test the following hypotheses:

**Hypothesis** **1.** Individual-level vertical trust is positively linked with SE, i.e., (a) job satisfaction, (b) health, and (c) job performance.

**Hypothesis** **2.** Group-level teamwork is positively linked with SE, i.e., (a) job satisfaction, (b) health, and (c) job performance.

**Hypothesis** **3.** Group-level transformational leadership is positively linked with sustainable employability, i.e., (a) job satisfaction, (b) health, and (c) job performance.

## 2. Materials and Methods 

Ethical approval for this project, including all data collections, was obtained from the Stockholm local ethics committee (ref no. 2015/857-31/5). Informed consent was obtained from all study participants in connection with all data collections. Three waves of a surveys (November/December 2015, June/July 2016, and November/December 2016) among healthcare employees were conducted. The participants were employed at the Stockholm regional healthcare organization, which offers a wide range of care (e.g., primary, psychiatric, rehabilitation, and acute hospital care). The employees were recruited to the study because their managers participated in a leadership training intervention [26] aimed at increasing implementation leadership skills. The surveys were a part of the training, as each manager received feedback concerning his or her leadership behaviors. The constructs measured among employees across these 3 timepoints included evaluations of implementation-specific factors (e.g., changes in procedures, readiness for change), evaluations of psychosocial workplace factors (e.g., leadership, job insecurity), and self-ratings of individual outcomes (e.g., work engagement, self-rated health).

### 2.1. Procedure and Participants

The sample consisted of all subordinates for the 54 managers who participated in the intervention [26]. The subordinates (*N* = 1136) received a link to the questionnaires at the three time points (referred to as T1, T2, and T3 hereinafter) through their work email addresses. They also received information about the study and information about their right not to answer or to prevent the data from being used in research and about how the data would be handled (e.g., anonymity). Two reminders were sent, and managers whose units had a response rate lower than 70% received an additional reminder to encourage their subordinates to complete the questionnaire. Of the total subordinate pool, we investigated only the data that was gathered from the nonmanagerial staff. Table 1 summarizes the demographics of the study samples across the measurement times as well as the panel sample consisting of employees who participated in all the measurements. The analyses were conducted among employees who completed the questionnaires at all three time points, leaving a sample of 269 individuals working in 42 units. A dropout analysis was conducted (χ2 test for gender, independent-samples Mann–Whitney U tests for quantitative variables) to compare our sample with the dropouts and those excluded from the analyses. The analysis concerned demographic data, predictors, and the outcome variables at T1. The results from the dropout analyses showed that those in the final sample had longer tenure (8.76 vs. 7.87; *p* = 0.005) and reporter better teamwork (3.88 vs. 3.72; *p* = 0.029) than those excluded and those who dropped out. The final sample did not significantly differ from those excluded or from the dropouts regarding any other variables.

### 2.2. Measurements

For all the scales (except for job performance, see below), a 5-point answer scale, in which the response alternatives ranged from 1 (*strongly disagree*) to 5 (*strongly agree*), was used.

*Vertical trust* was measured with a four-item subscale from the Copenhagen Psychological Questionnaire, second edition, (COPSOQ II) [35]. The subscale measures an individual’s trust regarding the organization and its conduct (e.g., “I can trust the information that comes from the management”; α = 0.90).

To measure *Teamwork,* four items based on Taylor and Bowers’s scale [36] were used. These items assess group processes in a professional setting to indicate whether a team works well together and how it structures its work tasks (e.g., “At my workplace we solve problems in a constructive way”; α = 0.89).

*Transformational leadership* was measured using seven items from the Global Transformational Leadership Scale [37]: “My manager communicate(s) a clear and positive vision of the future” and “My manager is clear about his/her values and practices what he/she preaches” (*α* = 0.82). The employees were asked to rate their agreement with these statements about their immediate manager.

To measure *Job satisfaction,* three items developed by Hellgren, Sjöberg, and Sverke [38] were applied. This scale measured the extent of happiness and satisfaction with one’s work (an example item is “I enjoy my work”; *α* = 0.93).

*Health* was measured with one item from the COPSOQ II [35]: “In general, I would say my health is good.”

*Job performance* was measured using three items from a workplace productivity scale [39]. The scale consisted of items concerning the amount, the quality, and the efficiency of work (e.g., “How would you describe your efficiency at work in the last week?”; α = 0.93), and its response alternatives ranged from 1 (*low*) to 10 (*high*).

### 2.3. Design

**Figure 1:**
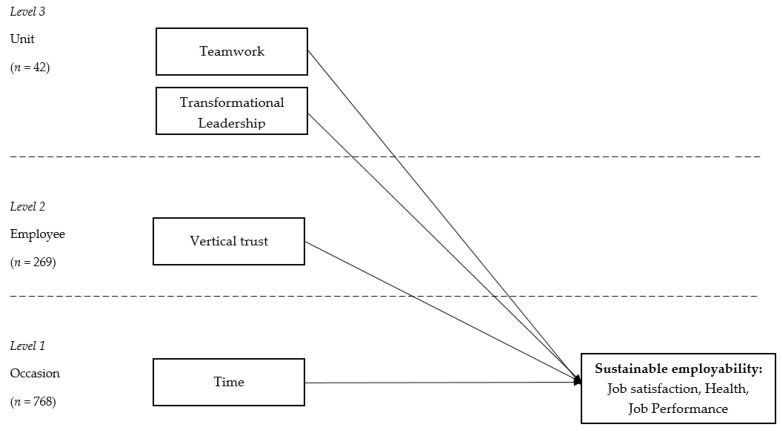
Conceptual model and measurement levels.

The data had a three-level structure, i.e., 768 measurement occasions were nested within 269 employees who were then nested in 42 units. Figure 1 represents the conceptual model and data structure.

## 3. Results

### 3.1. Aggregation

Teamwork and transformational leadership were assessed by individual employees but treated as unit-level variables. This approach assumes that individuals within a unit share a common reality regarding teamwork and leadership. If this assumption is valid, we will find that ratings from respondents in the same unit are similar to one another and that they are more similar to one another than they are to ratings from other units [40]. We examined this expectation using the average interrater agreement coefficient (r_wg_; [41]) and the intraclass correlation coefficient (ICC[1] and ICC[2]; [40]). Median r_wg_ values were above 0.70 for both variables, suggesting that employee ratings within a given unit were reasonably consistent with one another. One-way analyses of variance suggested that respondents’ ratings of teamwork and transformational leadership differed significantly between units (*F*s > 4.00; *p*s < 0.001). Furthermore, ICC(1) values were 0.21 for teamwork and 0.24 for transformational leadership, suggesting that approximately 20% of the variance in ratings was due to unit membership. Finally, the reliability of the group means, as measured by the ICC(2) coefficient, was 0.76 for teamwork and 0.79 for transformational leadership. Together, these analyses demonstrated sufficient homogeneity of within-unit variance and supported the aggregation of individual responses to create unit-level variables for teamwork and transformational leadership.

### 3.2. Preliminary Analyses

Table 2 and Table 3 show the means, standard deviations, and correlations of the study variables. In Table 2, correlations below the diagonal represent occasion-level correlations, and correlations above the diagonal represent between-person correlations (employee level). Table 3 displays correlations at the unit level.

Given the three-level model, we calculated ICCs as a proportion variance at the second and third levels [42]. The intraclass correlations (ICCs) for job satisfaction were 0.19 at Level 2 and 0.07 at Level 3, showing that approximately 19% of the variance in job satisfaction could be attributed to variations that occur between employees, while 7% could be attributed to variations between units. The ICCs for health (0.64 and 0.01, respectively) showed that 64% of the variance in health is explained by between-person differences, and 1% is attributed to units. Finally, the ICCs for job performance were 0.38 at Level 2 and 0.07 at Level 3. Approximately 38% of the variance in job performance could be explained by variations that occur between employees and 7% by variations that occur between units. We can conclude that the variance in these variables is due to occasion differences, individual differences and group differences. The ICCs were relatively small at the unit level. However, because nesting has a substantial impact on the inferences [42], we decided that a three-level multilevel analysis is the most appropriate here.

### 3.3. Hypotheses Testing 

To address the problem of the influence of distinct levels of social job resources on sustainable employment, we conducted separate multilevel analyses for each dependent variable, i.e., job satisfaction, health, and productivity. The analyses were performed using HLM software [43]. In each analysis, we started with a three-level intercept-only model with no predictors to decompose the total variance in three terms and to benchmark the model fit and explained variance. Next, in Model 1, we introduced time to control whether the outcome changed over the measurement occasions. In the next step, we introduced all the social job resources present in our investigation, i.e., Level 2 variable, vertical trust, and Level 3 variables, teamwork and transformational leadership. For a meaningful interpretation of the intercept, predictors were grand-mean centered, and time was recoded as follows: T1 was coded as 0, T2 was coded as 1, and T3 was coded as 2. We report the results at the 0.10 level of significance to ensure that we do not miss potentially interesting findings given the relatively low number of units (*N* = 42) and participants (*N* = 269). 

Table 4 presents the results of the multilevel analysis with job satisfaction as an outcome. Model 0 is a three-level intercept-only model for job satisfaction. In Model 1, we entered time as an occasion-level variable (Level 1) to control whether job satisfaction changed over the course of the year. After entering this variable, the overall model fit improved, i.e., deviance decreased; ∆D (1) = 45.92, *p* < 0.001. Time negatively predicted job satisfaction: γ = −0.25, SE = 0.04, *p* < 0.001. Therefore, job satisfaction decreased by 0.50 over the course of the year. Next, we added social job resources (Model 2), which significantly improved the model fit: ∆D (3) = 37.99, *p* < 0.001. Vertical trust was a positive level-2 predictor of job satisfaction: γ = 0.23, SE = 0.05, *p* < 0.001. Individuals who rated their vertical trust higher expressed higher job satisfaction across measurement occasions than those who rated their vertical trust lower. This result supports Hypothesis 1a. While both teamwork (γ = 0.16, SE = 0.14, *p* = 0.28) and transformational leadership (γ = 0.12, SE = 0.09, *p* = 0.18) were positively linked with job satisfaction, these relationships were not statistically significant. In Model 2, the amount of variance explained at the occasion level (Level 1) was 9.46%; at the employee level (Level 2), it was 5.26%, and the that proportion explained the variance at the unit level (Level 3) was 85.71%.

Table 5 presents the results of the multilevel analysis with self-rated health as an outcome. Model 0 is a three-level intercept-only model for health. After entering time as a predictor in Model 1, the overall model fit did not improve: ∆D (1) = 0.07, *p* = 0.79. Health did not significantly change across measurement occasions: γ = −.01, SE = 0.02, *p* = 0.80. In Model 2, we added social job resources. This addition significantly improved the model fit: ∆D (3) = 13.03, *p* = 0.005. Vertical trust positively predicted health: γ = 0.14, SE = 0.06, *p* = 0.021. Individuals high (vs. low) in vertical trust rated their health as better across measurement occasions. This result is in line with Hypothesis 1b. Teamwork was a positive predictor of health: γ = 0.32, SE = 0.16, *p* = 0.046. Units with better teamwork had more healthy employees across measurement occasions. This result supports 2b. Transformational leadership did not significantly predict health: γ = −0.08, SE = 0.09, *p* = 0.396. The amount of variance explained at the employee level (Level 2) was 3.77%, and the proportion of explained variance at the unit level (Level 3) was 1%.

Finally, to investigate the influence of distinct levels of social job resources on job performance, we conducted a three-level multilevel analysis with job performance as a dependent variable (see Table 6). Model 0 is a three-level intercept-only model for job performance. After entering time as a predictor in Model 1, the overall model fit did not improve: ∆D (1) = 0.17, *p* = 0.68. Job performance did not significantly change across measurement occasions: γ = −0.02, SE = 0.04, *p* = 0.67. In Model 2, we added social job resources. This addition significantly improved the model fit: ∆D (3) = 17.58, *p <* 0.001. Vertical trust positively predicted job performance: γ = 0.28, SE = 0.08, *p <* 0.001. Across measurement occasions, individuals high (vs. low) in vertical trust assessed their job performance as better. This result is in line with Hypothesis 1c. Teamwork was a positive predictor of productivity: γ = 0.45, SE = 0.24, *p* = 0.07. Units with better teamwork had employees reporting that they are more productive across measurement occasions. This result supports Hypothesis 2c. Contrary to 3c, transformational leadership was a negative predictor of productivity: γ = −0.26, SE = 0.15, *p* = 0.09. A higher result by 1 unit in the manager’s aggregated transformational leadership means 0.26 lower productivity. The amount of variance explained at the employee level (Level 2) was 8.62%, and the proportion that explained the variance at unit level (Level 3) was 37.50%.

## 4. Discussion and conclusion

### 4.1. Theoretical Contributions

The aim of the current study was to investigate if social resources can promote SE over time. Based on the definition of SE from van der Klink et al. [3], we operationalized SE as productivity, health, and job satisfaction, to capture employees’ ability to work productively while retaining health and well-being. Furthermore, in this study, vertical trust, team work, and transformation leadership were chosen as social resources, operating on different levels and different planes, furthering the JD-R theory [16]. Overall, the findings demonstrated that social job resources have a role in shaping employee job satisfaction, health, and performance, partially supporting all three hypotheses. More specifically, distinct resources played different roles for these three facets of SE, with vertical trust having a positive impact on all three facets, teamwork on two, and transformational leadership lacking a positive impact. 

Our results showed that vertical trust, measured at the individual level, was important for all three facets of sustainable employment in healthcare. It had a positive effect on employee job satisfaction, health, and job performance over time. Vertical trust represents how trustful an employee is with regard to the information that comes from management as well as employees’ perceptions of organizational justice. Hence, vertical trust is based on fair treatment (top–down) from the company, which generates employee trust towards the organization (bottom–up). Thus, this resource follows a vertical axis. According to the JD-R theory, vertical trust as a social job resource may trigger the motivational process and facilitate the achievement of goals at work. Our results corroborate other findings that link social capital with job well-being at work [29], trust with job performance [30], and justice with health [44].

Another social job resource we investigated in this study was teamwork, i.e., the extent of the collaborative effort of the group members to achieve a common goal. The similarity of individual perceptions of group processes within units allowed us to analyze this resource at the group level. This aggregation made it possible to compare healthcare units with each other and investigate how the levels of teamwork in different units affect individual employees in these units. The results showed that, in units high in teamwork, individual employees reported better productivity and health, though there was no statistically significant impact on job satisfaction. First, this result might imply that good collaboration and communication among team members facilitate task execution. Medical care is a team effort [32]; therefore, efficient cooperation translates into higher performance. Our research demonstrated that the influence of teamwork on productivity extends beyond individual perceptions. We showed a cross-level relationship in which unit-level teamwork affected individual performance; thus, we demonstrated that a work environment rich in good teamwork is beneficial for individual job performance. Next, the results also demonstrated that units with better teamwork have more healthy members than other units. Good teamwork signifies collaboration and support that can work as buffer in times of stress [45], helping employees lower the psychological cost of their job demands. Indeed, in his meta-analysis, Halbesleben [46] demonstrated that, after seeking social support at work, employees are less exhausted, which suggests that social resources are functional in dealing with stress. Given the link between stress and health (e.g., frequency of infections; [47]), our result shows that teamwork may shield against the negative consequences of stressors on health in the workplace. Moreover, positive team collaboration may lead to the development of strong social bonds among team members. Multiple studies to date have linked the quality of social relationships with people’s health and life expectancy [47,48]. Thus, our research shows that units with a good teamwork climate benefit individual employees: in such workplaces, employee health is better.

Finally, we also examined how the leader-team dyad affects SE. Because each unit had its own leader and the leaders’ ratings within the group showed similarities, we treated transformational leadership as a group-level vertical social job resource. The results demonstrated that transformational leadership did not allow us to predict individual job satisfaction or health; thus, the third hypothesis was not supported. One possible explanation for this result may be the relative importance of this construct compared to teamwork and vertical trust: by controlling for the resources that are provided by other team members and individual trust towards the organization, leadership may be less central to individual satisfaction and health. In fact, while both leader–member exchange (LMX) and team–member exchange (TMX) are associated with workplace outcomes, such as job performance or job satisfaction, a recent meta-analysis [49] demonstrated that TMX shows incremental validity above and beyond that of LMX for some outcomes (including job satisfaction). This result shows the distinctiveness of these variables but may also imply that they have separate roles for distinct employee outcomes, as we have observed here. Another possibility relates to the power that our study had to detect this potential relationship: with only 42 healthcare units, it is probable that a low-strength relationship was less likely to be detected.

Contrary to our hypothesis, transformational leadership was linked with lower productivity. Units in which managers displayed higher levels of transformational leadership than the managers of other units displayed lower individual job performance among followers than other units. This result largely contradicts previous studies, where transformational leadership in general is positively related to individual-level follower performance (for a meta-analysis see: [50]). We reanalyzed our data to examine the sole influence of transformational leadership on job performance (with vertical trust and teamwork excluded from the model), and we found that, on its own, transformational leadership is indeed a significant and positive predictor of job performance. The change in the influence of transformational leadership on job performance upon the inclusion of the other variables in the model may represent a case of a ‘suppression effect’, where a variable becomes a more efficient predictor of the criterion when another variable is included [51,52]. Transformational leadership, vertical trust, and teamwork may all have reciprocal relationships. In this case, when other influences are controlled by adding vertical trust and teamwork to the equation, then the direct effect of transformational leadership is seen to increase to a more genuine level. However, because we did not hypothesize such a link, the mechanism explaining the possible negative link between transformational leadership and job performance should be investigated in future research. 

To sum up, this paper makes three contributions to the existing literature. First, we investigate the role of distinct levels of social job resources for SE in healthcare. Although scholars recognize the complexity of organizational life, i.e., the fact that companies comprise different structural or hierarchical levels, job resources (as well as demands) have mostly been investigated at the individual level [53]. While models that focus on the individual level have been valuable, it is still necessary to understand and model group (i.e., contextual) factors that affect an individual’s functioning in the workplace [54]. By acknowledging the common psychosocial work environment that employees share in their units, we can more accurately capture the complexity of organizational phenomena and develop more sophisticated theoretical models. SE itself is a multilevel phenomenon that may have predictors located at many levels of the workplace. Thus, we extend the previous literature by exploring social job resources located at both the individual and group levels. Second, we examine how social job resources operate in distinct planes of organizational life, i.e., how they are exchanged in both vertical and horizontal axes. Including these different directions is important because it allows us to capture the meaning of organizational, managerial, and peer influences in the workplace. Finally, while the meaning of social support for employee well-being and organizational behaviors has received wide attention from scholars [55], and in the healthcare setting [56], here, we further the theory on other social job resources, i.e., vertical trust, teamwork, and transformational leadership, and their link with distinct facets of SE. While many studies addressed the importance of the factors that we chose here [29,30,31,32,33,34], to the best of our knowledge, no previous attempt was made to understand their joint effects as ‘social job resources’ and thus their relative importance in determining SE. Understanding which factors are more important to create SE is important from a practical view as it may indicate which goals interventions should target.

### 4.2. Practical Implications

The implementation of policies and practices aiming at developing staff’s employability is a valuable strategy to ensure employees’ commitment and productivity over time. Productivity, health, and well-being have been found to be related reciprocally [7,57]; thus, finding factors such as social resources that improve the quality of work and working conditions for employees’ health and well-being can ultimately equate to finding ways to achieve the high job performance that healthcare organizations strive for. Combined with other research [15], our study points to the importance of building a pool of social job resources in the healthcare setting as a means of increasing SE. Thus, we propose that facilitating the creation of a resourceful work environment through top–down redesign strategies, leadership processes or human resource management and human resource development practices should be an important organizational aim. 

More specifically, our results point to the importance of vertical trust in shaping all facets of employability, and thus, underline the role of integrity and fairness in the organizational conduct. The quantity as well as quality of organizational communication may be one mean to increase vertical trust [58]. The information that is conveyed should provide not only clarity but also reinforce predictability of the work situation [59]. When employees observe coherence between organizational communication and actual practice, employees tend to trust information from the management. Trust is especially important in healthcare, where changes aimed at optimizing patient care are introduced frequently. The uncertainty related to the change process may make employees especially attuned to the matters of fairness [60,61]. Managers should ensure accountability with clear performance standards that apply equally to everyone. Thus, our results call for following procedural, informational, and interpersonal justice rules [62] as means of increasing SE. Moreover, alignment of the information given with organizational actions might be an important strategy to maintain or increase vertical trust in the long run [63]. 

Next, our research demonstrates the relevance of promoting good teamwork to enhance individual health and performance at work. Teamwork is particularly relevant in healthcare given that patient care is a group effort [32]. Coordination can be facilitated by providing appropriate channels of communication and ensuring the existence of project management supportive tools among the team members with clear divisions of tasks and deadlines. Teams could also learn about constructive feedback, problem-solving strategies, and ways of overcoming interpersonal conflicts. To provide an adequate context for teamwork, an organizational incentive system and a leadership that requires and rewards teamwork should be in place to facilitate teamwork in the long run.

Interestingly, transformational leadership demonstrated having a less profound role for employee job satisfaction and health than teamwork. This pattern of results suggests the need to ensure that social job resources can be exchanged in a horizontal direction, i.e., between teammates, because these resources may be more readily available and accessible, and thus exert a stronger impact on employee health and well-being. Therefore, programs that allow employees to develop their social skills are warranted. Another practical implication may involve individuals being proactive in reaching for social job resources, e.g., developing high quality social connections [64].

Finally, the present study demonstrates that it is vital to acknowledge the level at which social resources function to understand and, in the long run, modify employee outcomes through designing and implementing interventions in the workplace that target the level in which these resources operate: at the individual, group or organizational level. [65]. In a recent review on interventions to promote sustainable employability, seven different studies were identified [66]. However, the majority of interventions were delivered at the individual level, e.g., where employees participated in individual training sessions with a physical therapist [67]. Based on our finding that vertical trust and teamwork affect SE over time, interventions on the group or even organizational level might be more fruitful to increase SE. In addition to targeting the source on the appropriate level, interventions at the group or organizational level also have the benefit of reaching larger groups of employees in a structured and uniform way by targeting the organization, management, and design of work [68]. Examples of these kinds of organizational interventions are training that target team-based burnout, teamwork, or implementation leadership [26,69,70]. 

### 4.3. Limitations

Despite the contribution this paper makes to research on social job resources and employment sustainability, we must note several limitations of this study. First, our study measures were based on self-reports. These declarative and one-source data may be subject to bias due to common method variance (CMV) [71]. To reduce this possibility, predictors have been aggregated over measurement occasions, and teamwork and transformational leadership have also been aggregated across the units to eliminate individual variance in group-level measures while maintaining the variability of the outcome variables. These precautions decrease but do not preclude the possibility that CMV affected the pattern of results in this study. Therefore, future research might include distinct measurement methods (e.g., objective performance reports) or sources of information (e.g., colleagues).

Next, the fact that the measurements were made during an active intervention may raise concern about the influence that the intervention itself had on the relationships we observed. One may argue, for instance, that social job resources might become especially relevant in times of stress [55]. If this is the case, then the time of organizational change may have elevated the importance of social job resources as predictors in our study. However, it is important to note that it is hardly ever the case that nothing truly happens in the organization for a year. In fact, healthcare in Sweden is a workplace in which organizational changes and interventions occur on a regular basis: continuous changes to employees’ work practices are introduced so that patients are able to receive updated and high quality care [72]. Therefore, the measurements we made happened in healthcare’s natural environment, and we believe that the shape of the results was not affected by the ongoing intervention. Moreover, the managers were trained in implementation-specific skills, and this was not directly the focus of our study variables.

In the current study, we solely focused on the motivational process as described by the JD-R model and a subset of job resources (i.e., social job resources) to predict SE. While this choice was justified given the previously discovered role of social job resources for staff retention among healthcare workers [15], future research should develop our findings using other types of resources such as organizational (e.g., learning opportunities) or psychological resources (e.g., self-efficacy) to predict SE. In addition, the JD-R model [16] proposes that apart from the motivational process, there is a simultaneous impairment process, wherein taxing job demands lead to employee exhaustion. While our research focused on factors promoting excellent work ability, for a fuller picture future studies should integrate both processes when investigating predictors of SE in healthcare.

Finally, this study focuses on a healthcare sample, which is a female-dominated sector in all Organisation for Economic Co-operation and Development (OECD) countries [73]. Future studies should replicate our results in other countries as well as test if the relations of social resources and SE are similar in other female-dominated sectors, such as education.

### 4.4. Future Directions

SE occurs when employees are able to continue working productively and making valuable contributions throughout their lives while retaining their health and well-being [3]. This conceptualization led us to differentiate three components of SE: productivity, health, and well-being. Researchers also argue that—apart from positive attitudes, job motivation, and psychological well-being—SE requires having the right competences for one’s work [3,66,74]. Thus, given the long-term perspective of SE, employees should also possess the capability to continuously learn and develop their competences [74,75]. Future research may, therefore, investigate to what extent social resources may be beneficial to learning at the workplace that leads to sustainable employability. For one thing, knowledge sharing is a behavior in a team that may inspire such development [76], and research demonstrates that trust is critical for knowledge sharing in teams [77]. Similarly, team climate and empowering leadership are linked with individuals’ knowledge-sharing behaviors [78]. Research should also examine other types of social job resources that may be specifically focused on learning and investigate their role for SE. For instance, mentoring may be viewed as a vertical top–down social job resource aimed at developing capabilities for future roles. It would be worth exploring to what extent mentoring opportunities allow the long-term goal of SE to be achieved. 

In future research in this area, attention should be given to identifying what kind of organizational culture and leadership styles promote the presence and exchange of social job resources in the workplace. For example, a manager’s emotional intelligence may play a role in incorporating the focus on relations (and not just tasks) in the workplace [79], and thus may promote the development of social job resources in that unit. Another important avenue would be to identify which social job resources are actually malleable and to what extent. This will enable HR practitioners and managers in the healthcare sector to apply evidence-based initiatives. 

Organizational decision-makers may face a dilemma between stimulating workers’ employability and retaining them [80]. Indeed, employees who are highly skilled may be motivated to find a new employment outside the organization if they are dissatisfied. It is possible, however, that the social job resources we investigated may act as “pull” factors affecting employees’ intentions to stay, because they satisfy the need for affiliation and relatedness. Social factors are among the most common influencers of staff retention [15]. Future research could, thus, inspect the joint effects of organizational climate that emphasizes development for employability along with building social ties to examine whether the presence of social job resources can protect against actual turnover for highly employable workers.

Further, the focus of SE is a long-term perspective, i.e., future employability of employees regardless of how close they are to retirement. Future research should focus on investigating how social job resources are related to the length of working careers and, for example, if they can prevent early retirement. As loneliness often occurs among the elderly [81], an opportunity to cultivate social bonds may be an important motivator to keep working. 

SE is a mutual responsibility of both organizations and individuals. While organizational-level interventions have been acknowledged as optimal to tackle the problem of stress in the workplace [82], it has simultaneously been recognized that employees themselves can also actively shape the demands and resources of their jobs to better adjust them to their preferences [83]. This employee-driven solution to job redesign has been named job crafting. Through job crafting, employees can alter the characteristics of their jobs to answer their need for optimal levels of job demands and job resources. In fact, one type of job-crafting behavior is ‘increasing one’s social job resources’ [84]. This goal can be achieved by, e.g., asking colleagues for help with task execution or seeking manager feedback about one’s competencies. Therefore, employees can proactively introduce the proper amount of social job resources to their environment as a bottom–up form of intervention in the workplace. Similarly, research points to the possibility of team crafting [85,86] by which employees collaboratively shape their demands and resources to achieve their group goals. Given these multiple potential ways to introduce optimal amounts of social job resources in the workplace, future research should investigate how top–down and bottom–up interventions that target social job resources can jointly affect the work environment in healthcare.

Social job resources should be introduced in a way that makes them functional to both employee well-being and organizational aims. If, at some point, interpersonal relations become more important than, e.g., the quality of work or productivity, the organizational goals may suffer. For instance, a psychological phenomenon called ‘groupthink’ [87] describes a dysfunctional situation that occurs within a group in which the desire for group harmony and a tendency to avoid conflict result in irrational decision-making outcomes [88]. It is therefore important to introduce productive forms of teamwork, where team members are open to discussing opposite points of view and deal with conflicts in an effective way. Furthermore, some individuals may not be motivated to seek more social job resources (e.g., supervisory feedback) to balance job demands and perform better but to satisfy their egos. In fact, Roczniewska and Bakker [89] showed that employees who score high in narcissism tend to seek social job resources as a way to boost their self-esteem; because they strive for admiration, narcissists want this self-love to be reinforced by others [90]. Therefore, when these individuals ask for feedback and guidance, they may actually unnecessarily consume a leader’s time, which is counterproductive. Are there any other downsides to an abundance of social job resources in the workplace? This topic seems understudied, and thus, we encourage future researchers to explore this phenomenon.

Achieving SE is a timely challenge for the healthcare sector in many countries. In this paper, we demonstrated that social job resources that operate at distinct levels and in different planes of organizational structure can substantially affect how satisfied, healthy, and productive employees are in these settings. By acknowledging the multiple levels and directions at which social job resources operate, we build a better understanding of the complexities of organizational life, and we can introduce occupational health interventions that address problems at the right level.

## Figures and Tables

**Table 1:** Demographics for the employees’ sample at each measurement occasion (T1-T3).

	Measurement Occasion			
	T1	T2	T3	T1-T3
Number of healthcare units	54	45	45	42
Number of participants (% women)	849 (87%)	524 (89%)	480 (90%)	269 (89%)
Age *M* (*SD*)	47.23 (11.62)	47.14 (11.42)	48.33 (10.94)	48.25 (10.60)
Tenure *M* (*SD*)	8.16 (8.03)	7.83 (7.49)	8.58 (7.77)	8.73 (7.69)
Permanent position (% of sample)	89%	90%	92%	93%
Full-time employment (% of sample)	70%	72%	71%	72%

**Table 2:** Intercorrelations between the study variables (Level 1 and 2).

Variable	M	SD	1	2	3	4	5
1. Vertical trust	3.23	0.93	—	—	0.37 ***	0.19 **	0.22 ***
2. Time ^1^	—	—	—	— ^2^	— ^2^	— ^2^	— ^2^
3. Job satisfaction	3.96	0.99	—	−0.20 ***	—	0.25 ***	0.34 ***
4. Health	4.20	0.92	—	−0.00	0.20 ***	—	0.23 ***
5. Job performance	8.09	1.29	—	−0.00	0.24 ***	0.21 ***	—

*Note.* ICC: Intraclass correlation coefficient. ^1^ Coded: 0 = Time 1, 1 = Time 2, 2 = Time 3. ^2^ Time averaged within a person is a constant; therefore, no correlation statistics are computed for between-person analyses with time variable. Correlations at the occasion level (*N* = 768) are displayed below the diagonal. Correlation at the employee level averaged across 3 occasions are displayed above the diagonal (*N* = 269). The correlational analyses do not account for the nested structure of the data. *** *p* < 0.001; ** *p* < 0.01.

**Table 3:** Intercorrelations between the study variables (Level 3).

Variable	M	SD	1	2	3	4	5
1. Teamwork	3.90	0.42	—				
2. Transformational leadership	3.95	0.61	0.54 ***	—			
3. Vertical trust	3.23	0.61	0.52 ***	0.68 ***	—		
4. Job satisfaction	4.00	0.43	0.50 **	0.57 ***	0.50 **	—	
5. Health	4.23	0.42	0.19	0.28	0.18	0.35 *	—
6. Job performance	8.07	0.59	0.42 **	0.09	0.23	0.26	0.02

*Note.* Correlations at unit level (*N* = 42) are averaged across 269 employees and 768 occasions. The correlational analyses do not account for the nested structure of the data. *** *p* < 0.001; ** *p* < 0.01; * *p* < 0.05.

**Table 4:** Multilevel analysis predicting job satisfaction from time (Level 1), trust (Level 2), and teamwork and transformational leadership (Level 3).

	Model					
	0		1		2	
*Fixed part*						
	Par. Est.	SE	Par. Est.	SE	Par. Est.	SE
Intercept	3.98 ***	0.06	4.23 ***	0.07	4.22 ***	0.05
Level 1−Occasion						
Time			–0.25 ***	0.04	–0.25 ***	0.04
Level 2−Individual						
Vertical trust					0.23 ***	0.05
Level 3−Unit						
Teamwork					0.16	0.14
Transformational leadership					0.12	0.09
*Random part*						
Variance decomposition						
Occasion	0.74		0.67		0.67	
Individual	0.19		0.21		0.18	
Unit	0.07		0.07		0.01	
*Model fit*						
Deviance (D)	2134.94		2089.02		2051.03	
Number of estimated parameters	4		5		8	
∆ D (M_n-1_)			45.92 ***		37.99 ***	
∆ parameters (M_n-1_)			1		3	

*Note.* Time is coded as T1 = 0, T2 = 1, T3 = 2. Vertical trust, teamwork, and transformational leadership are grand-mean centered. Par. Est = Parameter estimate. M_n_ = model number. *** *p* < 0.001, ** *p* < 0.01, * *p* < 0.05.

**Table 5:** Multilevel analysis predicting health from time (Level 1), trust (Level 2), and teamwork and Table 4.

	Model					
	0		1		2	
*Fixed part*						
	Par. Est.	SE	Par. Est.	SE	Par. Est.	SE
Intercept	4.20 ***	0.05	4.20 ***	0.06	4.19 ***	0.05
Level 1—Occasion						
Time			–0.01	0.02	–0.01	0.02
Level 2—Individual						
Vertical trust					0.14 *	0.06
Level 3—Unit						
Teamwork					0.32 *	0.16
Transformational leadership					–0.08	0.09
*Random part*						
Variance decomposition						
Occasion	0.30		0.30		0.30	
Individual	0.53		0.53		0.51	
Unit	0.01		0.01		0.00	
*Model fit*						
Deviance (D)	1751.43		1751.36		1738.06	
Number of estimated parameters	4		5		8	
∆ D (M_n-1_)			0.07		3.85 **	
∆ parameters (M_n-1_)			1		2	

*Note.* Time is coded as T1 = 0, T2 = 1, T3 = 2. Vertical trust, teamwork, and transformational leadership are grand-mean centered. Par. Est = Parameter estimate. M_n_ = model number. *** *p* < 0.001, ** *p* < 0.01, * *p* < 0.05.

**Table 6:** Multilevel analysis predicting productivity from time (Level 1), trust (Level 2), and teamwork and transformational leadership (Level 3).

	Model					
	0		1			2
*Fixed part*						
	Par. Est.	SE	Par. Est.	SE	Par. Est.	SE
Intercept	8.09 ***	0.08	8.11 ***	0.09	8.09 ***	0.09
Level 1—Occasion						
Time			–0.02	0.04	–0.02	0.04
Level 2—Individual						
Vertical trust					0.28 ***	0.08
Level 3—Unit						
Teamwork					0.45 ^†^	0.24
Transformational leadership					–0.26 ^†^	0.15
*Random part*						
Variance decomposition						
Occasion	0.92		0.92		0.92	
Individual	0.63		0.63		0.58	
Unit	0.11		0.11		0.08	
*Model fit*						
Deviance (D)	2441.33		2441.14		2423.56	
Number of estimated parameters	4		5		8	
∆ D (M_n-1_)			0.17		17.58 ***	
∆ parameters (M_n-1_)			1		3	

*Note.* Time is coded as T1 = 0, T2 = 1, T3 = 2. Vertical trust, teamwork, and transformational leadership are grand-mean centered. Par. Est = Parameter estimate. M_n_ = model number. *** *p* < 0.001, ** *p* < 0.01, * *p* < 0.05, ^†^ *p* < 0.10.
